# Microbiological and Physicochemical Evaluation of Hydroxypropyl Methylcellulose (HPMC) and Propolis Film Coatings for Cheese Preservation

**DOI:** 10.3390/molecules29091941

**Published:** 2024-04-24

**Authors:** Vanessa B. Paula, Luís G. Dias, Letícia M. Estevinho

**Affiliations:** 1 Doctoral School, University of León (ULE), Campus de Vegazana, 24007 León, Spain; 2 Centro de Investigação de Montanha (CIMO), Instituto Politécnico de Bragança, 5300-253 Bragança, Portugal; ldias@ipb.pt (L.G.D.); leticia@ipb.pt (L.M.E.); 3 Laboratório Associado para a Sustentabilidade e Tecnologia em Regiões de Montanha (SusTEC), Instituto Politécnico de Bragança, Campus de Santa Apolónia, 5300-253 Bragança, Portugal

**Keywords:** cheese, microbiological analysis, physicochemical parameters, films, HPMC, propolis, phenolic compound variation

## Abstract

Dairy products are highly susceptible to contamination from microorganisms. This study aimed to evaluate the efficacy of hydroxypropyl methylcellulose (HPMC) and propolis film as protective coatings for cheese. For this, microbiological analyses were carried out over the cheese’ ripening period, focusing on total mesophilic bacteria, yeasts and moulds, lactic acid bacteria, total coliforms, *Escherichia coli*, and *Enterobacteriaceae*. Physicochemical parameters (pH, water activity, colour, phenolic compounds content) were also evaluated. The statistical analysis (conducted using ANOVA and PERMANOVA) showed a significant interaction term between the HPMC film and propolis (factor 1) and storage days (factor 2) with regard to the dependent variables: microbiological and physicochemical parameters. A high level of microbial contamination was identified at the baseline. However, the propolis films were able to reduce the microbial count. Physicochemical parameters also varied with storage time, with no significant differences found for propolis-containing films. Overall, the addition of propolis to the film influenced the cheeses’ colour and the quantification of phenolic compounds. Regarding phenolic compounds, their loss was verified during storage, and was more pronounced in films with a higher percentage of propolis. The study also showed that, of the three groups of phenolic compounds (hydroxybenzoic acids, hydroxycinnamic acids, and flavonoids), hydroxycinnamic acids showed the most significant losses. Overall, this study reveals the potential of using HPMC/propolis films as a coating for cheese in terms of microbiological control and the preservation of physicochemical properties.

## 1. Introduction

Cheese is the most widely consumed dairy product. It is usually made via the fermentation of pasteurised milk. Depending on the production process, it can be hard, semi-hard, or soft. Cheeses can be classified according to their water activity (*a*_w_) and pH levels [1,2]. These physicochemical properties can be measured quickly and easily and are important for ensuring the microbial safety of a food product. Dairy products include milk, cheese and yoghurt. They are nutrient-rich sources of protein, calcium, vitamins, minerals, and water, making them particularly susceptible to microbial spoilage. Lipids and proteins present in these products can catalyse chemical reactions that alter the taste and quality of dairy products [3,4]. The microbial degradation of cheese during storage is one of the most important issues in the food industry [5,6]. The absence of a protective barrier, such as packaging, leads to high moisture loss in some cheeses, resulting in increased hardness and organoleptic property loss [7]. Since the acceptance of food by the final consumer depends on organoleptic properties, including aroma and flavour [8], it is important to use packaging to prevent the chemical, physical, biochemical, and microbiological degradation of the cheese, extending its useful shelf life and enhancing its overall quality [6]. Usually, cheese is packaged in plastic packaging. This is not biodegradable, causing a major environmental problem [9]. Therefore, it is necessary to reduce the use of plastics in food packaging and use natural, edible substances. Currently, most research has focused on creating edible food packaging with antimicrobial and antioxidant properties that inhibit the growth of microorganisms, reduce oxidative spoilage and water loss, and maintain microbial food quality [3,10,11]. Edible packaging can be applied by dipping, spraying, electrostatic spraying, and brushing in the case of coatings and via individual wrapping in the case of films [12,13]. Natural substances commonly used to manufacture biodegradable films include polysaccharides, proteins, lipids, and specific compounds (derived from animals, plants, or microbes) [3]. Polysaccharides are abundant in nature, degradable, and non-toxic, and have been shown to provide a good oxygen and flavour barrier and display excellent properties in terms of strength. Cellulose is the most abundant polysaccharide in nature, and the most commonly used derivatives in the formation of edible films are methylcellulose (MC), hydroxypropyl methylcellulose (HPMC), and carboxymethylcellulose (CMC) [14,15]. Among these derivatives, HPMC is the most widely used for edible packaging, drug capsules, and drug release systems due to its adequate water solubility and biodegradability, excellent film-forming ability, and ideal mechanical and barrier properties [16]. However, as most polysaccharides are hydrophilic, the resistance of films decreases under conditions of high humidity. This disadvantage can be overcome by adding hydrophobic substances such as essential oils and waxes [3]. In addition to essential oils, other natural products such as plant extracts [17] and bee products have been highlighted for use in foods as natural preservatives [18,19,20]. Several studies have reported using propolis as a food preservative and active packaging [21]. It can be applied to the surface of foods or incorporated into food formulations [22]. Numerous studies have been carried out to investigate the use of propolis to preserve cheeses. Tumbarski et al. [23] investigated the potential to improve the quality and extend the shelf life of Bulgarian Kashkaval cheese by using three different ethanolic extracts of propolis in combination with the polymer carboxymethylcellulose. On the other hand, El-Deeb et al. [24] studied the use of different concentrations of propolis as a natural preservative in the production of Kareish cheese in order to evaluate its efficacy. A study by Guirguis et al. [25] investigated the potential of using propolis extracts in processed cheese spreads to improve both shelf life and decontamination. Propolis is a complex natural resinous mixture collected by *Apis mellifera* L. bees from the components of various plant segments, exudates, and buds, which are then combined with pollen and salivary enzymes secreted by the bees [26,27]. It is a chemically very complex beekeeping product, consisting of flavonoids and phenolic acids and their esters, waxes, essential oils, pollen, and various organic compounds [28,29]. The composition of propolis contributes to its biological activities, such as antibacterial [30], anti-inflammatory [31], antitumour [32], cytotoxic [33], and antioxidant activities [30], among others. Due to these properties, propolis has been incorporated into applications centered around gelatine-based biopolymer matrices [34,35], chitosan [36,37], hydroxypropyl methylcellulose [38], and starch [39], among other uses. Thus, encapsulation technology has proven to be a promising solution in terms of improving the use of bioactive propolis compounds in the pharmaceutical and food industries [40,41,42]. Its utilization prevents lipid oxidation, extends the shelf life of food products, and improves visual quality [20,43,44]. Indeed, several studies have included propolis in different food formulations, including fruits, vegetables, juices, meats, seafood, dairy products, and others [43,45,46,47,48].

This study aimed to evaluate cheese preservation by applying a film onto its surface using a propolis formulation based on the HPMC polymer and studying the variation in phenolic compounds present in the HPMC/propolis film during the storage time. This research adds significantly to our current knowledge by investigating changes in phenolic compound composition during cheese storage and assessing the impact of this loss on different phenolic groups (hydroxybenzoic acids, hydroxycinnamic acids, and flavonoids).

## 2. Results

The experimental design included the study of the impact of HPMC film propolis content (factor 1) and storage days (factor 2) on the dependent variables: microbial growth (total mesophilic, yeasts and moulds, lactic bacteria, total coliforms, *Escherichia coli* and *Enterobacteriaceae*), and physicochemical parameters (pH, *a*_w_, colour, cheese weight, and phenolic compounds).

### 2.1. Microbiological Analysis

The results of the microbial analysis indicate that the cheese samples were not fit for human consumption, especially at the time of purchase (T_0_), as the microbial counts were quite high for all microorganisms tested (Table 1). The results show that, of the five initial samples of cheese tested (batch sampling n = 5), four were positive for *E. coli*, including at values higher than those established by Regulation (EC) 1441/2007 (log CFU/g = 3). Coagulase-positive *Staphylococcus*, *Listeria monocytogenes*, *Clostridium* spores, and *Salmonella* spp. were not detected in any of the tested cheeses.

**Figure 1:**
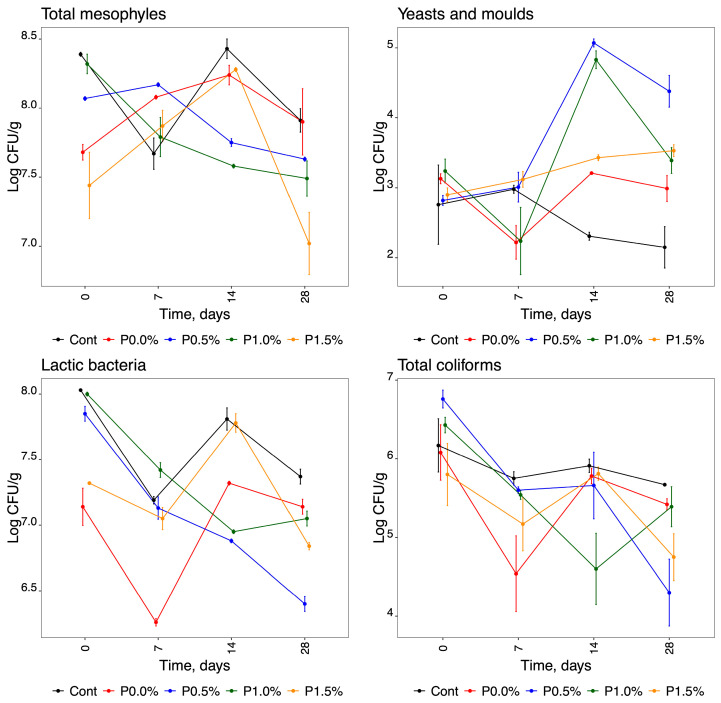

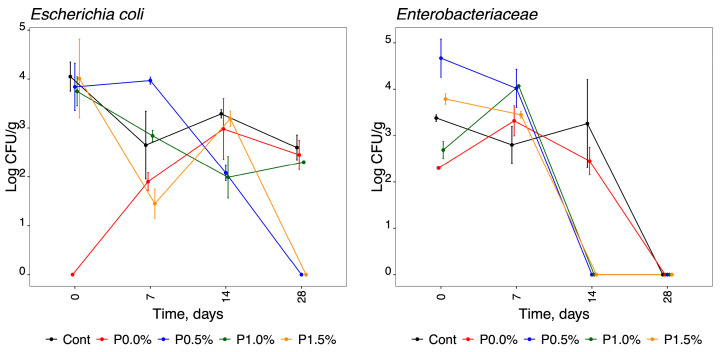
Graphical representation of the behaviour of each microorganism analysed in cheese samples for the different parameters studied over time: total mesophyles; yeasts and moulds; lactic bacteria; total coliforms; *Escherichia coli*; and *Enterobacteriaceae*.

Table 2 presents the ANOVA data obtained for the microbiological parameters. The models obtained are significant, with coefficients of determination generally higher than 0.93 (errors lower than 0.38), indicating that the models can explain at least 93% of the original variability of the experimental data. These results showed a good fit, except for the total coliforms, which showed a poor linear fit (low R^2^ value = 0.767; high residual standard error [RSE = 0.790]). In all ANOVA models, the interaction term between the two factors was significant (values < 0.001). All dependent variables failed at least one of the assumptions (see Table 2). Therefore, considering that the use of ANOVA was suboptimal for addressing departures from data ideality, PERMANOVA was employed to assess the significance of the terms. In this analysis, the interaction term between the two factors was significant for all the dependent variables, meaning that the data should be evaluated using the graphs shown in Figure 1 to assess the effects of factor 1 (microbiological analyses) and factor 2 (time in days) when interacting with different variables (Table 2).

Looking at Figure 1 and the graphs representing each of the microorganisms tested, each parameter shows a distinct behaviour. The total number of mesophilic bacteria in the cheese samples analysed was, on average, 7.98 ± 0.41 log CFU/g at time zero (T_0_). It can be observed from the graph relating to total mesophiles that most of the cheeses had different initial microbial loads, with values between 7.44 and 8.39 log CFU/g. However, all cases showed a slight decrease in the total number of mesophilic bacteria from T_0_ to T_28_, with an average of 7.59 ± 0.36 log CFU/g at T_28_. At the final time (T_28_), the parameter P1.50% was found to have a lower value, followed by P1.0%, P0.5%, and finally P0.0% and the control, all of which had very similar values. From the analysis of the graph of yeast and mould counts, we found that, in cheese coated with propolis (P0.5%, P1.0% and P1.5%), the count of these microorganisms increased during storage, with average values of 2.98 ± 0.22 and 3.77 ± 0.54 log CFU/g for T_0_ and T_28_, respectively. Contrary to the total mesophilic count, in this case, the control and P0.0% parameters gave lower final counts of yeasts and moulds. Notably, the filamentous fungi were only verified in T_0_ for all propolis concentrations.

The number of lactic bacteria (Figure 1) decreased with time, with average values of 7.67 ± 0.41 and 6.96 ± 0.37 log CFU/g for T_0_ and T_28_, respectively. Overall, the total coliform count (Figure 1) decreased over storage, with average values of 6.25 ± 0.36 and 4.67 ± 1.45 log CFU/g for T_0_ and T_28_, respectively. It should be noted that there was only a progressive decrease in these microorganisms over the storage time in the cheeses coated with 0.5% propolis, with a lower count at T_28_ (2.15 ± 3.04 log CFU/g). At T_0_, the number of *E coli* (Figure 1) was high in four of the five analysed cheeses, with an average value of 3.91 ± 1.75 log CFU/g. In these products, this indicator of faecal contamination was absent at T_28_. In contrast, P0.0% did not show *E. coli* at T_0_, with a progressive increase occurring during storage. At T_0_, *Enterobacteriaceae* counts (Figure 1) ranged between 2 and 5 log CFU/g. In all cases, these bacteria gradually declined during storage. In fact, in products coated with films containing propolis, *Enterobacteriaceae* were absent after 14 days of storage, but in the other cheeses evaluated, this behaviour was only observed after 28 days.

### 2.2. Determination of pH, Water Activity, and Weight Loss

The results obtained for the physicochemical properties, pH and *a*_w_, as well as the variation in cheese weight over time for the different samples studied, are shown in Table 3.

**Figure 2:**
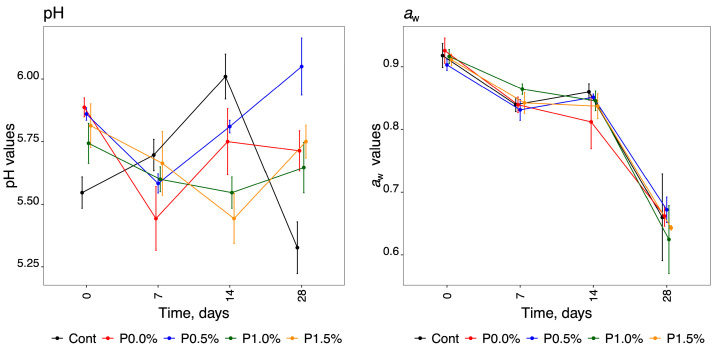
Graphical representation of the results obtained for pH and *a*_w_ over the storage time of the cheeses for the five HPMC formulations.

Applying a two-factor ANOVA with a significant interaction term to these two physicochemical parameters revealed that the interaction terms were statistically significant (*p*-value < 0.001). The ANOVA models exhibited significance for both analysed parameters (*p*-value < 0.001), yielding R^2^ values of 0.9770 and 0.9930 for pH and *a*_w_, respectively. These values indicate that the model explains 97.70% and 99.30% of the variability in the experimental data for pH and *a*_w_, respectively. However, only the pH results demonstrated normal and homogeneous variances. Although the *a*_w_ results displayed homogeneity of variances (Levene test *p*-value = 0.550), they did not meet the assumption of normality (Shapiro–Wilk test *p*-value < 0.001). In this context, PERMANOVA was also employed to confirm the significance of the interaction terms. This result further supports the notion that the pH and *a*_w_ data should be interpreted using the graph presented in Figure 2. The pH of the samples had no trend, as seen in Figure 2. Each parameter behaved differently, but in general all showed a decrease in pH from T_0_ to T_28_ (mean values of 5.75 ± 0.15 and 5.61 ± 0.19 for T_0_ and T_28_, respectively), except for P0.5%, which increased at T_28_ (5.86 for T_0_ and 6.05 for T_28_). Overall, the pH values varied between 5.33 and 6.05 for the different parameters over the storage time, with a mean value of 5.69 ± 0.19.

Water activity (*a*_w_) values decreased with storage time for all the conditions tested (Figure 2, graph of *a*_w_ values). There was a decrease from 0.91 ± 0.005 to 0.65 ± 0.013, with mean values obtained for T_0_ and T_28_, respectively.

Regarding the weight of cheeses, there was a decrease during the period of cheese storage in the refrigerator. The average weight of the cheeses was 97.22 ± 4.78 g at T_0_ and 78.36 ± 5.04 g at T_28_, representing an average loss of 20.49 ± 1.85 g between the two periods analysed.

There was no correlation between the three parameters being studied, although there was a trend between *a*_w_ values and cheese weight. Indeed, both decreased during storage.

### 2.3. Colour Determination

**Figure 3:**
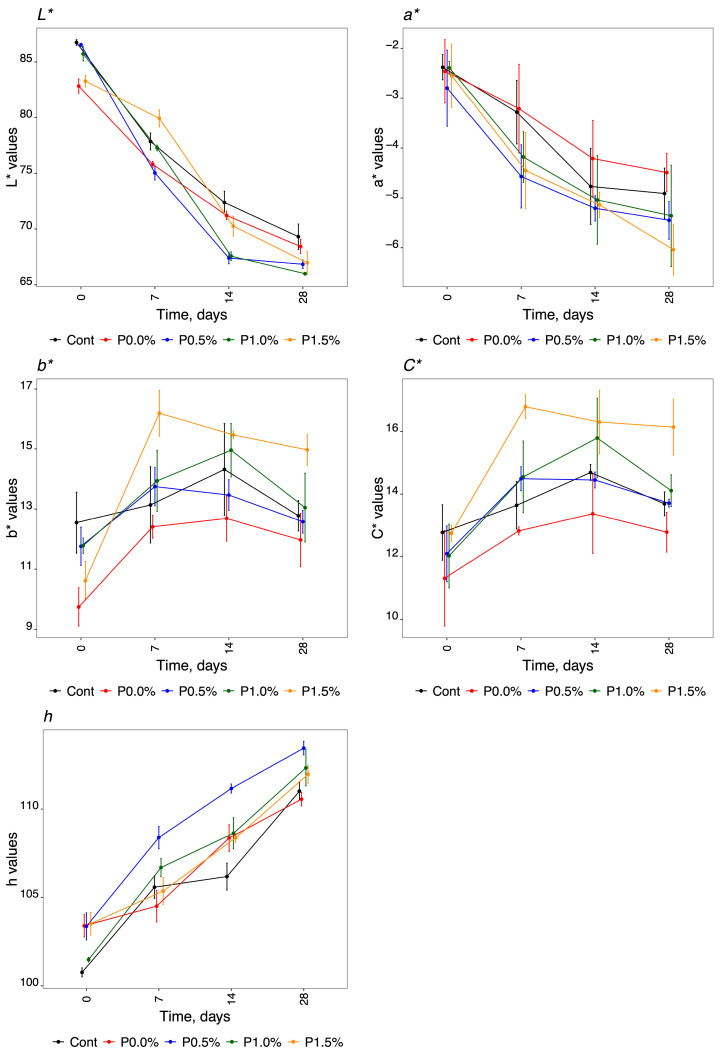
Instrumental colour analysis evaluating the CIELAB method and cylindrical coordinates for the different parameters during storage time. L*, luminosity; a* and b* chromaticity coordinates; h, hue, and C*, chroma.

The results obtained when using the CIELAB method to analyse the colour of the cheese samples of the experimental design, established for the study of HPMC film composition with propolis and storage days, are shown in Figure 3.

Regarding instrumental colour analysis, the cheeses showed similar behaviour over the storage time for the five parameters evaluated (L*, a*, b*, h and C*). The lightness (L*) was higher at T_0_ for all the different HPMC compositions analysed, with a mean value of 85.01 ± 1.85. As for the storage time, luminosity decreased for all the formulations and the control, reaching a mean value of 67.51 ± 1.33 at T_28_. These results demonstrated that L* was not significantly reduced for the samples treated with propolis compared to the control. The a* colour coordinates were negative towards green, with mean values of −2.52 ± 0.17 and −5.25 ± 0.59 for T_0_ and T_28_, respectively. At P0.0% and in the control, the decrease was not as pronounced as it was in the formulations that contained propolis in the coating. Indeed, at the final time (T_28_), these two different HPMC compositions had fewer negative values than the others, meaning that these cheeses did not obtain a green colour. The b* chromaticity coordinates were greater than zero, with mean values ranging from 11.29 ± 1.10 to 13.07 ± 1.13 for T_0_ and T_28_, respectively. The P1.5% formulation presented higher b* values over the storage period; consequently, these cheeses became more yellow than the others. The P0.0% formulation had lower b* values at all storage times. The cylindrical coordinates, represented by chroma (C*) and hue (h°), increased with the storage time for all the products analysed. We obtained mean values of 12.19 ± 0.60 for C* and 102.51 ± 1.29 for h at T_0_, and mean values of 14.09 ± 1.25 and 111.86 ± 1.13 for C* and h, respectively, at T_28_. Although the behaviour of the cheeses was similar for the C* values, we observed that the values obtained during storage for P0.0% were reduced in comparison with the formulations containing propolis. The other three formulations (control, P0.5%, and P1.0%) obtained intermediate values.

### 2.4. Quantification of Phenolic Compounds

The total phenolic compound (TPC) concentrations of the different formulations were determined spectrophotometrically using the Folin–Ciocalteu method. The concentrations of propolis which were added to the formulations containing HPMC ranged from 0.0 to 10%. For the determination of TPCs, we established a calibration line with gallic acid (GA) as the standard procedure. The results were expressed as mg gallic acid equivalents per gram of sample (mg GAE/g). The content of these compounds in the formulations containing HPMC was found to be directly related to the increase in the concentration of propolis added to the formulations. This relationship followed a linear trend in the range of 0 to 7.5%, with an acceptable correlation coefficient of 0.998 (available as Figure S1 and Table S1 in the Supplementary Material). The relationship was defined by the linear equation TPC (mg GAE/g) = 0.92(±0.02) × [propolis] − 0.11(±0.08). However, the result obtained using 10% propolis content showed a slight deviation (11.54 ± 0.31 mg GAE/g) from this linear trend, as evidenced by the increased errors present in the parameters of the linear equation (TPC (mg GAE/g) = 1.09(±0.06) × [propolis] − 0.38(±0.29)) and the lower correlation coefficient (R = 0.989) when this value was included.

### 2.5. Quantification of the Variation of Phenolic Compounds

**Figure 4:**
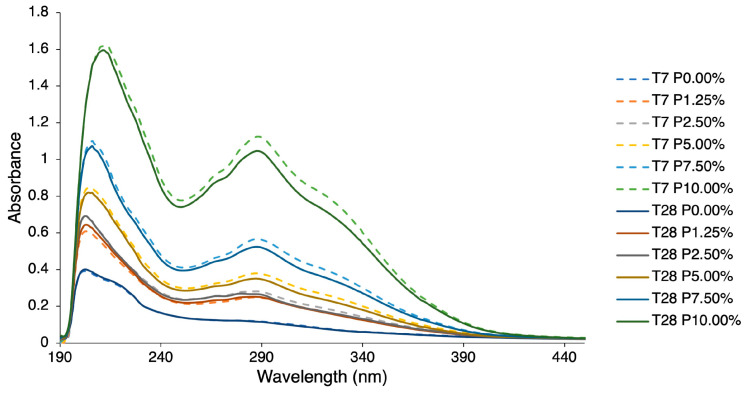
UV-vis spectra of different formulations of HPMC and propolis for T_7_ and T_28_.

Figure 4 shows the variation of TPCs over time (T_7_ and T_28_) for HPMC formulations containing 0.00, 1.25, 2.50, 5.00, 7.50 and 10.00% propolis. The quantification of the three classes of TPC [hydroxybenzoic acids (HBA), hydroxycinnamic acids (HCA), and flavonoids (FLAV)] used for the different formulations (P1.25, P2.50, P5.00, P7.50 and P10.00%) was carried out using standard mixed solutions of gallic acid, ferulic acid, and quercetin, as reported in Table 4.

From the analysis of Figure 4, it can be seen that the absorbance of spectra increased in direct proportion to the concentration of propolis added to the HPMC/propolis formulation. The values obtained for TPCs decreased slightly during storage time (T_7_ to T_28_).

These results are also shown in Table 4, which contains the outcomes when the concentrations of HBA, HCA, FLAV, and TPCs, obtained using the formulation without propolis (P0.00%, containing only the HPMC polymer), are subtracted from the results of formulations with propolis.

The assumptions about the ANOVA analysis were validated and we confirmed that the data showed homogeneity of variances (Levene’s test *p*-value > 0.05) and normality (Shapiro–Wilk test *p*-value > 0.05). Two-way analysis of variance (ANOVA) was performed, and the results showed a good overall fit (R^2^ > 0.994; residual standard error < 7.2). The results for HBA and TPCs showed no significant interaction terms, in contrast to those obtained for HCA and FLAV.

**Figure 5:**
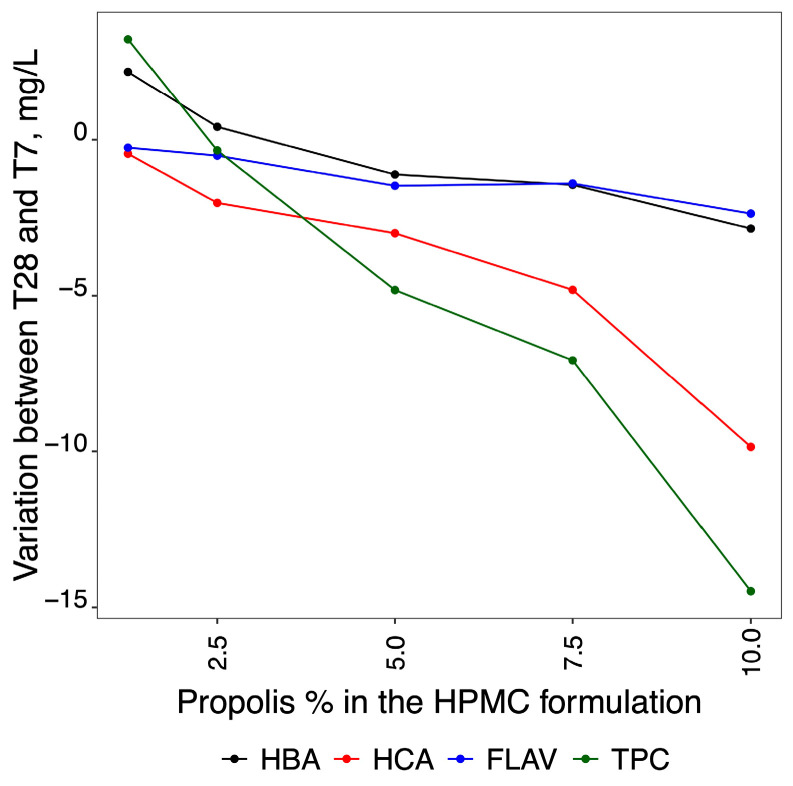
Graphical representation showing the losses observed between T_7_ and T_28_ for HBA, HCA, FLAV, and TPCs.

The HBA results only showed significant variability for factor 2 (amount of propolis in the film), indicating that the variation in the quantified amount of propolis did not change between the two storage times (T_7_ and T_28_). However, the ANOVA results for TPC data showed significant differences for both factors, i.e., the storage times and the amount of propolis incorporated in the film (represented by different letters). These differences indicate that the TPCs varied according to the storage times. These variations are illustrated in Figure 5. Although the concentrations at FLAV are relatively low, Figure 5 might initially suggest a similar trend to that of HBA. However, closer inspection reveals that they are more consistent with the behaviour observed for HCA, which is confirmed by the results of the two-way ANOVA analysis.

The result of the subtraction of the difference in the HBA, HCA, FLAV, and TPC values between the two times studied (T_7_ and T_28_) is shown in Figure 5.

Figure 5 also shows that the loss of TPCs is directly proportional to the concentration of propolis added to the film. Of the three TPC classes, HCA shows the greatest loss over the storage time for all propolis films. The films with the lowest propolis content (P1.25% and P2.50%) showed the lowest TPC losses, although the TPC concentration, over time, remained constant in the formulation containing 2.5% propolis and increased slightly in the products coated with 1.25% propolis. The variation in TPC concentrations was due to the additive variation of the three phenolic classes analysed (HBA, HCA, and FLAV).

## 3. Discussion

### 3.1. Microbiological Analysis

Dairy products provide an ideal environment for the proliferation of microorganisms that can act as vectors for spoilage microorganisms and foodborne pathogens [49,50]. Although the pasteurisation of raw milk is a critical technological step that significantly reduces the number of microorganisms, it does not result in a completely sterile product [49]. In microbiological studies of milk and dairy products, it was found that *E. coli*, *Salmonella* spp., *L. monocytogenes*, *S. aureus*, yeasts, and moulds are the main spoilages or pathogenic microbiota found in these products [51,52]. Therefore, the risk of infection and foodborne illness from consuming milk and dairy products tends to increase in relation to the duration of storage [53]. There are several stages in cheese production at which microorganisms can be introduced into the product. Mature cheeses are ready for human consumption after a certain storage period under specific temperature, humidity, and ventilation conditions that allow characteristic physical and chemical changes to occur [54].

The microbiological analyses carried out on the cheeses in this study revealed high levels of total aerobic mesophilic bacteria, yeasts, and moulds, total coliforms, *E. coli* and *Enterobacteriaceae*, particularly at the initial time (T_0_). This result was likely related to the hygienic conditions in which the milk was obtained, as well as the processing, the storage, and the transportation of both the raw materials and the final product [55]. Regulation (EC) 1441/2007 [56] does not set limit values for total aerobic mesophilic bacteria, yeasts, and moulds in cheeses because the microbiota responsible for fermentation can be incorrectly quantified when these microbiological parameters are evaluated. However, it is crucial to assess yeasts and moulds since these microorganisms can cause economic losses due to sensory changes in products and may pose a risk to public health because some mould species produce mycotoxins [5,57]. According to INSA values (interpretation of microbiological test results in ready-to-eat foods and on surfaces in the food preparation and distribution environment) [57], off-flavours can occur when lactic acid bacteria exceed 8 log CFU/g, Gram-negative bacteria exceed 7 log CFU/g, and yeasts exceed 6 log CFU/g. This can lead to product spoilage due to acid and gas production [58]. In our study, although many of these indicators were found in some cases, their levels did not exceed the thresholds indicating off-flavours [57]. The results for total mesophiles mainly showed lactic acid bacteria with an average count of less than 8 log CFU/mL, while the results for yeasts were consistently less than 6 log CFU/mL (as shown in Table 1). In cheeses coated with HPMC and propolis at concentrations of 0.5, 1.0 and 1.5%, there was an increase in the number of yeasts detected during storage under refrigeration conditions. The yeasts present in the products analysed showed an ability to multiply at low pH values, low temperatures, low water activity, and high-salt concentrations [59]. It should be emphasised that the yeasts present in cheese can have various origins and causes, including the quality of the milk, the processing environment, the fermentation process, and storage [59,60]. The yeasts present in the environment can easily adhere to the surface of cheeses and form a complex biofilm with other microorganisms [60,61], particularly in traditional aged cheeses. According to Fröhlich-Wyder et al. [61], the requirements for yeast growth inside the cheese depend on the availability of oxygen (Crabtree effect). The Crabtree effect is very important from an economic point of view for technologies based on the use of yeast. For example, the positive Crabtree yeast, *Saccharomyces cerevisiae*, may not be ideal for use in biomass production due to its sensitivity to glucose and oxygen and its preference for fermenting, rather than breathing, high levels of glucose at low oxygen levels. The presence of negative Crabtree yeasts, such as *Kluyveromyces marxianus*, *Candida intermedia*, and *Debaryomyces hansenii*, increases towards the end of ripening due to the ability to respire, even in the presence of high levels of glucose and low levels of oxygen, and to utilise residual lactose and/or galactose [62,63]. This suggests the possibility of *K. marxianus* growth on film-coated cheese despite oxygen limitations. In the control group, yeasts (*S. cerevisiae*) predominated, carrying out the metabolism of glucose and lactic acid, as well as the deamination or decarboxylation of amino acids in the corresponding products [64,65]. In coated products, as the conditions are not favourable for the growth of these yeasts, they may be viable but not cultivable [61].

The results obtained in this study are corroborated by the observations of Siripatrawan et al. [36]. These researchers observed that while the amount of propolis incorporated into chitosan films increased, *K. marxianus* was the dominant yeast in the cheese. Padilla et al. [66] reported that the yeast count in goat’s milk cheeses increased from 10^4^–10^5^ CFU/g at the beginning of ripening to 10^8^ CFU/g at the end of ripening. *K. marxianus*, *Yarrowia lipolytica*, *D. hansenii*, and *S. cerevisiae* are important in the ripening process of cheeses, giving them their characteristic flavours and texture through proteolytic activity and lactose fermentation [61,67].

In all experimental conditions tested, filamentous fungi were only present at T_0_. Cortés-Higareda et al. [68], Franchin et al. [69], Tumbarski et al. [23] and Pastor et al. [38] reported the effectiveness of propolis in inhibiting fungi; however, the control and P0.0% in the present study also showed no growth. Jia et al. [70] and Racchi et al. [71] reported similar results. These researchers observed that the growth of filamentous fungi was also affected by the *a*_w_ (fungi do not grow in water with activity values of less than 0.85) and pH values.

Mesophilic microorganisms are indicators of milk quality, reflecting the hygiene conditions it has been subjected to [57,72,73]. High levels of these microorganisms (>8 log CFU/g) are associated with non-compliance with good hygiene practices, the use of poor-quality raw materials, the breakdown of the cold chain, poor surface disinfection techniques, the occurrence of cross-contamination, faulty/insufficient heat treatment, and uncontrolled temperature/storage time and/or preservation [57]. The results obtained in this study for these microorganisms were always greater than 7 log CFU/g, i.e., higher than the level considered acceptable. However, since cheeses are fermented products, this number is overestimated as the total aerobic count includes lactic acid bacteria. According to Giannuzzy et al. [74], the acceptable level for fermented products is 6 log CFU/g. A level above 7 log CFU/g can affect the quality of the cheese and reduce its shelf life. The results showed that these microorganisms decreased from T_0_ to T_28_, particularly in cheeses coated with propolis. Studies carried out by Nessianpour et al. [75], El-Deeb et al. [24], Guirguis [25], and Yildirim et al. [11] have shown that different combinations of propolis extracts are effective against total mesophilic aerobic bacteria.

Lactic acid bacteria present in cheese and other dairy products play a crucial role in the ripening process and development of flavour [23]. However, they must be controlled as they can ferment and acidify products, causing off-flavours and other sensory changes. This feature makes them a good indicator of product freshness and shelf life [57,76]. The ratio of the total number of mesophilic and lactic acid bacteria is one indicator of product acceptability. This value should be less than 100 CFU/g in order for the product to be deemed safe [57]. The values obtained in our work for lactic acid bacteria were identical to those of the total counts of mesophilic aerobes, suggesting that most of the latter bacteria were lactic acid bacteria. The results show that the films containing propolis do not affect the number of lactic acid bacteria (values between 6.96 and 7.67 CFU/g) during the storage period compared to the control and the film without propolis. Yangilar et al. [77] also found levels of lactic acid bacteria between 6.90 and 7.89 log CFU/g in cheese samples coated with edible casein/natamycin films during storage.

Total coliforms are a sub-group of the Gram-negative *Enterobacteriaceae*. These are facultative anaerobes that ferment lactose in order to produce acid and gas. Coliforms are commonly found at low levels in raw milk, but pasteurisation effectively destroys these microorganisms in milk because they are not sporulated. However, poor hygiene, cold chain management and further processing (pasteurisation, cheese ripening) often reduce the detection of these issues in mature cheeses [78]. High counts of these microorganisms (>2.0 log CFU/mL) suggest failures in terms of general hygiene and/or inadequate heat processing temperatures [73]. Although there was a slight decrease in the expression of these microorganisms over the storage period, the results were always greater than 2 log CFU/g. Only the film with P0.5% propolis showed a decrease during the last storage period. These results suggest that the goat’s milk from which the cheeses were prepared was not adequately pasteurised or that the cheeses were not properly matured and stored. Moatsou et al. [79] state that coliforms can easily recontaminate cheeses through the environment.

*E. coli* is a public health threat as it is the most common cause of cheese-related foodborne diseases. Its presence in food indicates the possibility of faecal contamination and raises concerns about the possible presence of other microorganisms of faecal origin, including pathogens. Currently, *E. coli* is the most reliable indicator of faecal contamination among the commonly used faecal indicator organisms [80,81]. According to Regulation (EC) No. 1441/2007 [56], the legal limit for this microorganism is 3 log CFU/g. Some of the cheeses used in this work showed values that were higher than those considered acceptable, especially at the initial analysis time. However, at the last time (T_28_), the values presented were already within acceptable limits or absent. In our study, *E. coli* was absent at T_28_ in all formulations except for the cheese coated with HPMC and 1% propolis, suggesting that this natural product effectively destroyed this group of microorganisms. In fact, *E. coli* has a high stress tolerance to the acidity and low temperatures used in cheese ripening. It can survive in refrigerated and frozen foods [82]. Cosciani-Cunico et al. [83] observed the presence of *E. coli* in cheese.

*Enterobacteriaceae* are indicators of product hygiene, and their presence can be related to faecal contamination [84,85]. This group of organisms consists of Gram-negative bacteria, which are indicators of poor hygiene or contamination in dairy products after pasteurisation [86]. At the beginning of the analysis of the cheeses (T_0_), we found that they displayed a high presence of *Enterobacteriaceae*, meaning that the cheeses were contaminated after the pasteurisation of the milk. However, none of the samples analysed contained these microorganisms at T_28_. On the other hand, in cheeses covered with propolis-containing films, these microorganisms were no longer detected at the end of 14 days of refrigeration, indicating that propolis exerted antibacterial action against these microorganisms. Petrozzi et al. [87] also remarked that propolis can inhibit Gram-negative bacteria.

In the present study, all the samples analysed showed the absence of coagulase-positive *Staphylococcus*, *L. monocytogenes*, *Clostridium* spores, and *Salmonella* spp., indicating that cheeses can be considered satisfactory according to the standards established by Regulation (EC) 1441/2007 [56] for these parameters. However, as mentioned above, some cheeses were not fit for consumption because of *E. coli* concerns.

The cheeses used in our study are marketed with an average size of 100 g. Therefore, a different cheese was used for each formulation and test time, even if taken from the same batch. This approach may explain some of the variations observed, particularly in terms of the initial microbial loads. As seen from the five samples of cheese analysed initially, the regulations require that a batch is evaluated by considering a sample number of n = 5.

### 3.2. pH, Water Activity and Weight Loss

The importance of pH in food stability and preservation is well known. The term pH is equivalent to the logarithm of the concentration of hydrogen ions. The concentration of hydrogen ions in food is a controlling factor in the regulation of many chemical, biochemical, and microbiological reactions. In cheese, pH strongly influences ripening due to its effect on proteolysis [88]. In our study, variations occurred in pH values due to the variability of the natural product rather than the tested formulation since no clear trends were observed in relation to film type. This means that the addition of propolis to the film formulations did not affect the pH values of the cheeses during storage. Sun et al. [89] and Kaewprachu et al. [90] reported no significant differences in the pH values of food products when gelatine was added with food additives. Ibrahim et al. [91] also showed that propolis did not affect the pH of thyme labneh samples stored for 7 and 14 days. The pH values of the cheeses were found to agree with those reported for other Portuguese cheeses [92,93,94].

Water activity (*a*_w_) has emerged as one of the most important intrinsic factors in predicting the survival and proliferation of microorganisms in food as it directly impacts product stability and overall quality. It is important to note that the resistance of microorganisms to low *a*_w_ values is variable. In fact, some microorganisms can multiply at low *a*_w_ values, compromising the product’s safety. While most enzymatic reactions tend to slow down at *a*_w_ values below 0.80, it is worth noting that specific reactions can still take place at extremely low *a*_w_ values [95]. A decrease in pH was observed with storage time in all samples under investigation, regardless of the film used. The film had no effect on the level of water lost from the cheese. As a result of the loss of water from the cheeses, there was a loss of weight during storage. Pastor et al. [38] and Basch et al. [96] investigated the permeability of HPMC films to water vapour, and the results showed that these films had poor moisture barrier properties. However, the reduction in moisture in formulations containing HPMC contributed to an increase in tensile stress and the modulus of elasticity [96,97]. This led to us obtaining results that showed a significant reduction in the moisture content of cheese samples during the ripening process, regardless of the type of coating applied.

### 3.3. Colour

Colour can significantly impact product acceptance and commercial success, making it a critical parameter to consider in food packaging [91,98]. Lightness is lost during storage, with lightness values decreasing and moving away from 100 (L* = 0 black, L* = 100 white). This was found in all the cheeses studied. It was observed that the HPMC/propolis formulation with a higher percentage of propolis (P1.50%) obtained lower values of the chromaticity coordinate of a*, meaning that there was a colour change towards green, which intensified with the storage time of the cheese. The same phenomenon was observed in all experiments, with a colour change towards green over time. The slight increase in b* values over time meant that the colour became more yellow. The values obtained for C* (amount of saturation of colour) were not very high, meaning that the cheese samples had low luminosity in all HPMC formulations: they are dull. This result is supported by the values obtained for L*, which show that cheeses losy luminosity with conservation (decrease in values from T_0_ to T_28_). The values of h were close to 90°, which means that the colour of the samples was yellow and that this colour intensified with storage time, which was in agreement with the values obtained for the colour coordinates of b*.

The incorporation of propolis into the formulations with HPMC affected the colour values. These results corroborate the work of Khodayari et al. [99], who added propolis extract to a polymeric matrix of polylactic acid and found that the colour parameters differed from those of the control. Ibrahim et al. [91] showed changes in the colours of thyme labneh samples in terms of lightness (L*) and chromaticity coordinates a* (redness) and b* (yellowness) after the addition of propolis.

### 3.4. Phenolic Compounds

Phenolic compounds have a wide range of beneficial properties, including antioxidant, anti-allergenic, anti-inflammatory, anti-cancer, anti-hypertensive, and antibacterial effects. Polyphenols, which are divided into three classes (HBAs, HCAs and FLAV) based on their chemical structure, possess phenolic hydroxyl groups that have a strong affinity with protein binding, resulting in the inhibition of microbial enzymes and enhanced attachment to cytoplasmic membranes, thereby enhancing their antibacterial activity [100]. These groups contribute to bacterial cell death by helping to delocalise electrons, acting as proton exchangers and reducing the tendency of bacterial cells to adhere tightly to the cytoplasmic membrane, resulting in the leakage of cellular components from the cell [101,102]. Propolis is a well-studied natural product known to be rich in TPCs. The propolis sample used in this work has already been studied for its phenolic compound composition [103,104]. The quantification of phenolic compounds of different formulations of HPMC and increasing concentrations of propolis verified the existence of a direct relationship between these variables. Siripatrawan et al. [36] also found an increased presence of TPCs in propolis concentrations in chitosan films.

### 3.5. Variation of Phenolic Compounds over Time

Phenolic compounds are not completely stable and are easily degraded during storage, causing their biological properties to change [105]. We observed a decrease in the amount of TPCs with storage time. However, the results showed that, of the three classes of TPCs, HCAs displayed the most considerable losses over time. The decrease in phenolic compounds (p-coumaric and caffeic acids) over time was probably due to enzymatic oxidation [106]. Santos et al. [48] also found a decrease in the quantification of phenolic compounds in a propolis-incorporated yoghurt sample during refrigerated storage. Rzepecka-Stojko et al. [107] reported a decrease in the concentration of polyphenols in ethanol extracts of bee pollen that were stored for 12 months at different temperatures (refrigerated in the dark, kept at room temperature in the dark, and maintained at room temperature in sunlight). According to these researchers, refrigerated samples saw the most minor losses of these compounds. The oxidation reactions increase as the temperature rises [108].

Some authors [109,110] have observed that lactic acid bacteria decarboxylate HCAs into their vinyl derivatives, which means that HCAs lose the double bond in the side chain of the structure. According to Borges et al. [111], high concentrations of HCAs have antibacterial activity against a wide range of Gram-positive and Gram-negative bacteria. This means that the loss of this group of phenolic compounds during storage will impact cheese preservation.

## 4. Materials and Methods

### 4.1. Reagents and Samples

All reagents were of analytical grade and were used as they were purchased. Gallic acid (1-hydrate), obtained from Panreac (99%, Barcelona, Spain), was used as the standard. We acquired absolute ethanol (EtOH) from Panreac (HPLC quality, Barcelona, Spain, 99.9%) and diethyl ether from Carlo Erba reagents (brand), with both obtained in solvent form. Other reagents included the Folin–Ciocalteu reagent, obtained from Scharlau (Barcelona, Spain), and sodium carbonate (Na_2_CO_3_), acquired from Panreac (Barcelona, Spain). Hydroxypropyl methylcellulose (HPMC), with an average molecular numerical weight (Mn) of ~22 kDa and a viscosity of 40–60 cP (2% in H_2_O at 20 °C), was acquired from Sigma-Aldrich. Type II deionised water was used in all analytical work.

Cheese samples (n = 20) were purchased from a supermarket and vacuum-packed in pairs. These were mature goat’s cheeses (made from boiled goat’s milk, salt, calcium, and rennet) from the same production batch, and they had a total surface area of 117 cm^2^ and a weight of around 100 g per cheese. Cheese should be stored between 0 and 10 °C, as recommended by the manufacturer. The cheeses were analysed immediately after being purchased. They were stored at 4 °C throughout the 28 days of testing. The propolis sample was collected in Montesinho, a region located in the north of Portugal in the Trás-os-Montes sub-region. The sample was obtained by removing material from the panels of propolis traps.

### 4.2. Film Preparation

The ethanolic extract of propolis was prepared as described by Paula et al. [104]. Samples were prepared by mixing 5 g of raw propolis with absolute ethanol (1:5, *w*/*v*) and stirring the solution at 60 rpm overnight. The solution was then filtered through Whatman n° 4 filter paper and stored at −18 °C to remove the wax. The ethanol was then removed via evaporation using a rotary evaporator (IKA model RV8, VWR, Darmstadt, Germany). One hundred mL of diethyl ether and 100 mL of deionised water were added to the propolis extract obtained, resulting in the formation of two different visible phases. Diethyl ether was used to extract the maximum amount of phenolic compounds from the sample. The supernatant (diethyl ether) was transferred to a new container, and the solvent extraction process was repeated three more times until a clear boundary between the two visible phases appeared. From the resulting extracts, 0.2 g was weighed and dissolved in 10 mL of 80% absolute ethanol to produce the propolis solution used for film preparation.

Hydroxypropyl methylcellulose (HPMC, 2% *w*/*w*) was prepared in sterile deionised water and placed in a water bath at 50 °C for 2 h. The solution was then stirred (60 rpm) overnight at room temperature. The HPMC solution was then divided into equal parts in four sterile flasks, and the propolis solution was added. The propolis solution was added to the HPMC solution to give final propolis concentrations of 0.0, 0.5, 1.0 and 1.5% by weight of HPMC in the solution. The mixtures were emulsified at room temperature using a magnetic stirring plate (50 rpm). The dispersions that were intended for use in film formation were designated as P0.0% (film without propolis), P0.5% (film with 0.5% propolis), P1.0% (film with 1.0% propolis), and P1.5% (film with 1.5% propolis).

### 4.3. Cheese Application

The 20 cheese samples were randomly distributed among the trials of the experimental design. This experimental design involved studying four different time intervals (T_0_, T_7_, T_14_ and T_28_ days) using four applications of the HPMC/propolis formulation, and we included a control group for each time interval. In each experimental set-up, four cheeses were used, with each cheese corresponding to one of the specified time points. Random sampling was used to ensure that the results obtained from the control group could be considered indicators of the contamination levels in the remaining experimental trials.

The solutions, which were obtained by mixing HPMC and different concentrations of propolis (0.0, 0.5, 1.0, and 1.5%), were applied to 16 cheeses. Four cheeses were used for each HPMC/propolis formulation, and four other cheeses were used as the controls (20 cheeses in total). The control group, consisting of four randomly selected cheeses, did not use the HPMC/propolis formulation. These cheeses contained only the basic ingredients necessary for their production: boiled goat’s milk, salt, calcium, and rennet. The cheeses were analysed at four different points in time (0, 7, 14, and 28 days), and a different cheese was used at each time. The same cheese was used for microbiological and physicochemical analyses. It was ground in a mill (IKA Tube Mill, VWR, Darmstadt, Germany) under aseptic conditions and the quantity needed for the microbiological analyses was separated, leaving the rest for use in the physicochemical analyses. Three applications of the HPMC/propolis formulation were made to each cheese, with the sample allowed it to dry between each application to enhance the protective barrier on the surface of the cheese. The 20 cheeses were placed in a fridge at 2–4 °C.

#### 4.3.1. Microbiological Analysis

Microbiological analyses were conducted on days 0, 7, 14, and 28 of storage. Twenty-five grams of ground cheese samples were added with 225 mL of ringer’s solution and homogenised for 2 min (Stomacher 400, Seward, UK). After serial decimal dilutions using ringer’s solution, appropriate dilution samples (1 or 0.1 mL) were carefully applied or spread evenly on petrifilms or agar plates. All bacterial counts were quantified and expressed as log CFU/g, and each analysis was performed in duplicate.

Total mesophilic bacteria counts were enumerated on plate-count agar (PCA, AppliChem Panreac, Barcelona, Spain) by incubating samples at 30 °C for 48 to 72 h [112]. Yeast and mould enumeration was performed on Rose Bengal agar with chloramphenicol (Biolife, Bothell, WA, USA) and these were incubated at 25 °C for 48 h to 5 days to allow for fungi growth [113]. Lactic acid bacteria (LAB) were enumerated by plating on Man, Rogosa, and Sharpe (MRS) agar (Liofilchem, Roseto degli Abruzzi, Italy) and incubation at 30 °C for 48 to 72 h [114]. Total coliforms and *E. coli* were quantified using the SimPlate kit (Biocontrol^®^, Rakkestad, Norway) and these were incubated at 37 °C for 24 h [115]. *Enterobacteriaceae* were enumerated using Petrifilm™ *Enterobacteriaceae* Count plates (3M Health Care, St. Paul, MN, USA) and incubated at 37 °C for 24 h. To perform the detection of coagulase-positive staphylococci, Baird–Parker broth (VWR chemicals, Darmstadt, Germany) with Egg Yolk Tellurite (Himedia, Maharashtra, India) was incubated at 37 °C for 24 to 48 h [116]. The *Salmonella* 1-2 Test^®^ (Biocontrol) was used to detect *Salmonella* spp. in accordance with the official AOAC methods and the manufacturer’s instructions [117]. Rapid and reliable results were obtained within 16 to 20 h following pre-enrichment in buffered peptone water (25 g of sample was weighed and dissolved in 225 mL of peptone water). They were then incubated at 37 °C for 24 h. For the enumeration of sulphite-reducing *Clostridium* spores [118], 1 mL of the decimal dilutions was placed in a sterile tube. The solution was heat-treated at 80 °C for 10 min and then coated onto iron sulphite agar (Liofilchem, Roseto degli Abruzzi, Italy). These tubes were then incubated at 37 °C for 5 days. For the microbiological analysis of *L. monocytogenes*, 25 g of sample was homogenised for 2 min in 225 mL of Half Fraser Base CM0895 (Thermo Scientific, Oxoid, Göteborg, Sweden). The enumeration was performed according to a procedure adapted from ISO 11290-2:1998/Amd. 1:2004(E) [119]. After the incubation of the initial suspension at 20 °C for 1 h, 0.1 mL portions were surface-inoculated in duplicate on Oxoid Chromogenic Listeria Agar (OCLA, Oxoid) and incubated at 37 °C for 24 h. Samples that showed no growth were further analysed for *L. monocytogenes* using a method adapted from ISO 11290-1:1996/Amd.1:2004(E) [120]. The initial suspension was supplemented with SR 166 (Oxoid) and incubated at 30 °C for 24 h. It was then plated onto OCLA and incubated at 37 °C for 24 h. If no growth was observed, 0.1 mL of the same initial supplemented suspension was transferred to 10 mL of Fraser’s broth supplemented with SR 166 (Oxoid), incubated at 37 °C for 48 h, streaked onto OCLA, and then incubated again at 37 °C for 24 h.

The analyses for coagulase-positive, *Salmonella*, sulphite-reducing *Clostridium* spores and *L. monocytogenes* were only carried out at T_0_ and T_28_.

#### 4.3.2. Determination of pH, Water Activity and Cheese Weight

The pH was determined using a combined pH electrode connected to a digital pH meter HI8424 (Hanna Instruments, Gipuzkoa, Spain). After the cheese samples had been ground up, the pH value was measured directly from the sample. The result obtained was the arithmetic mean of three determinations. The water activity (*a*_w_) was measured via direct reading using the AquaLab equipment (Aqualab 4TE Decagon, Washington, DC, USA). The results recorded represent the arithmetic mean of three readings. Weight loss, expressed in grams, was monitored throughout the storage period.

#### 4.3.3. Colour Determination

The determination of colour was performed using the CIELAB (International Commission on Illumination) method, with the use of the Chroma Meter CR 400 colourimeter (Konika Minolta). Specifically, we evaluated the coordinates L*, a*, b*, C* and h, where −L* indicates darkness, +L* indicates lightness, −a* indicates greenness, +a* indicates redness, −b* indicates blueness, and +b* indicates yellowness. Further, the cylindrical coordinates’ h (hue) indicates the angle, with 0° indicating red, 90° indicating yellow, 180° indicating green, and 270° indicating blue. C* (chroma) is also an indicator, with C* = 0 indicating grey colour and C* > 0 indicating the purity or intensity of the colour (Hunter, LabScan XE, Reston, VA, USA).

### 4.4. Quantification of Phenolic Compounds

Total phenolic content (TPC) was determined in a microplate using the Folin–Ciocalteu method presented by Bobo-García et al. [121]. Aliquots of 20 µL of the HPMC/propolis formulation were mixed with 100 µL of Folin–Ciocalteu reagent (1:4 *v*/*v*) and shaken for 60 s in a 96-well microplate reader (Thermo Scientific Multiskan GO, VWR, Darmstadt, Germany). The mixture was allowed to stand for 240 s. Then, 75 µL of sodium carbonate solution (100 g/L) was added, and the mixture was shaken for 60 s. After 2 h at room temperature, the absorbance was measured at 750 nm using the microplate reader. The results were expressed as mg of gallic acid equivalents (GAE)/g sample, using a gallic acid calibration curve (20–250 mg/L).

This test was performed based on the HPMC/propolis formulations applied to the cheese and the formulations prepared for the phenolic compound’s variation test (methodology described below).

### 4.5. Quantification of the Variation of Phenolic Compounds over Time

For the phenolic variation test, different concentrations of propolis were used to prepare HPMC/propolis formulations to obtain final propolis concentrations of 1.25, 2.5, 5.0, 7.5, and 10.0% by weight of HPMC in solution. The formulations were placed in plastic Petri dishes and dried in a ventilated oven at 50 °C. After drying, the films were removed from the plate, and an area of 1.44 cm^2^ was cut out. The cut-out square of the film was placed in a test tube containing 4 mL of 95% absolute ethanol. The spectra were then read in the UV-vis spectrophotometer (VWR UV-3100PC spectrophotometer) in a wavelength range between 190 and 1100 nm using a quartz cuvette. The spectra were taken at 7 and 28 days after the film had been immersed in the solvent. The quantification of the phenolic compounds present in the solvent was determined using the calibration curves of gallic acid, ferulic acid, and quercetin from the work of Paula et al. [103].

### 4.6. Statistical Analysis

A two-way ANOVA (analysis of variance), also called two-factor ANOVA, was used to verify the influence of the composition of HPMC films (factor 1) and storage days (factor 2), as well as the effect of their interaction, on the growth of microorganisms or physicochemical parameters (such as responses and dependent variables). The ANOVA model was evaluated for its significance (considering a significance level of 0.05) and its coefficient of determination (R^2^ allows researchers to check the percentage of explanation of the original variability of the experimental data).

The normality assumption was checked by analysing the model residuals using the Shapiro–Wilk test. To assess the homogeneity of variances, the Levene’s test, which is less sensitive to departures from normality, was applied to several independent variables.

For dependent variables with normality problems or a lack of homogeneity of variances, non-parametric permutation-based MANOVA (often called PERMANOVA, i.e., multivariate analysis of variance based on distances and permutations) was used. This relies on dissimilarities to compare groups, similar to ANOVA, and is largely unaffected by heterogeneity in balanced designs. PERMANOVA uses permutations to compute F-statistics (pseudo-F), and the null hypothesis was that groups do not differ in the multivariate space of spread or position [122].

We used the open-source R software version’s R 4.2.2 GUI 1.79 High Sierra build (Mac OS) for data analysis. The libraries used were vegan [123], which allowed the application of PERMANOVA, and stats [124], which allowed the application of ANOVA and tests to assess the assumptions of ANOVA.

We used multiple and simple linear regression methods to assess the total content of hydroxybenzoic acids (HBA), hydroxycinnamic acids (HCA), and flavonoid (FLAV) compound groups. Additionally, we determined the TPC content along the UV spectrum using equations presented in a previous study [103].

Using multiple linear regression, we assessed the total hydroxybenzoic acids (results in mg of gallic acid per L of solution) using the following equation:

C_Gallic acid_ = 192 × Abs_275_ − 156 × Abs_345_ (1)

We determined the total hydroxycinnamic acids (results in mg of ferulic acid per L of solution) using the following equation:

C_Ferulic acid_ = 129 × Abs_325_ − 78 × Abs_380_ (2)

We determined the TPCs (results in mg of mixed phenolic compounds per L of solution) using the following equation:

C_TPC_ = 186 × Abs_220_ + 75 × Abs_345_ (3)

The analysis of flavonoids (results in mg of quercetin per L of solution) was carried out by using a simple linear regression to measure the solution’s absorbance at 375 nm. This procedure utilized the following equation:

C_Quercetin_ = 182 × Abs_375_ (4)

## 5. Conclusions

Dairy products demand strict adherence to procedures that are closely tied to various endogenous aspects of production, including raw materials, processing chains, ripening temperatures, water activity (*a*_w_), pH, environmental contamination, and operator practices.

The results presented in this study demonstrate that coating cheeses with HPMC/propolis formulations reduced the levels of total mesophilic bacteria, total coliforms, *Enterobacteriaceae*, and, in some instances, *E. coli* produced during cheese storage. This reduction can be attributed to the protective effects of the HPMC/propolis coating, which contains phenolic compounds that may inhibit bacterial growth. While a decrease in pathogenic bacteria was observed at the end of the refrigerated storage period, enhancing the transportation conditions of raw materials and better controlling storage conditions remain essential. Furthermore, the decline in physicochemical properties (pH and *a*_w_) over storage time also contributed to the microbiological control of the cheese samples.

The pH and *a*_w_ values of the cheeses during storage were not significantly affected by the addition of propolis to the HPMC film formulations.

Increasing the concentration of propolis in the formulations resulted in more yellow-coloured films compared to light-coloured HPMC films (P0.0%). This was a consequence of the pigments present in propolis. On the other hand, excess oxygen would cause browning because polyphenol oxidases use it to convert most phenolic compounds into brown pigments (melanins).

Our study showed that there was a direct relationship between the addition of propolis to HPMC formulations and the concentration of phenolic compounds in the resulting films. This meant that the HPMC/propolis formulation with a higher propolis content (P1.50%) provided better protection for the cheese samples. While phenolic compounds conferred a protective effect on food products due to their biological activities, there was a decrease in TPC concentrations with storage time, with HCAs showing the greatest losses. The results showed that the higher the percentage of propolis added to the formulations, the greater the loss of compounds over time. This observation suggests that there was an increased susceptibility to degradation by lactic acid bacteria as the concentration of propolis in the film increased.

In general, we observed a variation in the results of the different tests performed during the storage time of the cheese. This variation may be due to the use of independent cheese samples at each point in the study and/or uncontrolled variations in the film’s formation on the cheese’s surface.

Overall, the addition of propolis to HPMC film formulations can have variable effects on microbial growth, physicochemical parameters, and the colour of cheese samples during storage. Propolis showed potential antimicrobial properties, especially against certain microorganisms. The study also highlighted the need for proper pasteurisation and cheese ripening processes to ensure product safety and quality. These results provide valuable insights into the potential use of propolis-based films in cheese packaging, with consideration given to their effect on various quality parameters over time. However, propolis-based films would probably be more effective in foods that do not contain lactic acid bacteria, as these interact with the HCA groups of phenolic compounds and reduce their protective properties.

## Figures and Tables

**Table 1:** Effect and evolution of microbial growth (log CFU/g) over time in relation to different the concentrations of propolis applied to the cheese surface.

Microorganisms	Parameter	Days:	0	7	14	28
Total mesophiles *	Control		8.39 ± 0.01 ^ab^	7.67 ± 0.08 ^efgh^	8.43 ± 0.05 ^a^	7.91 ± 0.06 ^cde^
	P0.0%		7.68 ± 0.04 ^efgh^	8.08 ± 0.01 ^bcd^	8.24 ± 0.05 ^ab^	7.90 ± 0.17 ^cdef^
	P0.5%		8.07 ± 0.01 ^bcd^	8.17 ± 0.01 ^abc^	7.75 ± 0.02 ^efgh^	7.63 ± 0.01 ^efgh^
	P1.0%		8.32 ± 0.05 ^ab^	7.79 ± 0.10 ^defg^	7.58 ± 0.01 ^fgh^	7.49 ± 0.09 ^gh^
	P1.5%		7.44 ± 0.17 ^h^	7.87 ± 0.08 ^cdef^	8.28 ± 0.01 ^ab^	7.02 ± 0.16 ^i^
Yeasts and moulds *	Control		2.76 ± 0.40 ^efgh^	2.98 ± 0.04 ^cde^	2.31 ± 0.04 ^fgh^	2.15 ± 0.21 ^h^
	P0.0%		3.13 ± 0.05 ^cde^	2.22 ± 0.17 ^gh^	3.21 ± 0.01 ^cde^	2.99 ± 0.13 ^cde^
	P0.5%		2.82 ± 0.05 ^defg^	3.01 ± 0.15 ^cde^	5.07 ± 0.04 ^a^	4.38 ± 0.16 ^b^
	P1.0%		3.24 ± 0.12 ^cde^	2.24 ± 0.34 ^gh^	4.83 ± 0.09 ^ab^	3.39 ± 0.13 ^cd^
	P1.5%		2.90 ± 0.07 ^def^	3.12 ± 0.08 ^cde^	3.43 ± 0.03 ^cd^	3.53 ± 0.06 ^c^
Lactic bacteria *	Control		8.03 ± 0.00 ^a^	7.19 ± 0.02 ^ef^	7.81 ± 0.06 ^c^	7.37 ± 0.04 ^d^
	P0.0%		7.14 ± 0.10 ^f^	6.26 ± 0.02 ^i^	7.32 ± 0.01 ^de^	7.14 ± 0.04 ^f^
	P0.5%		7.85 ± 0.04 ^bc^	7.13 ± 0.06 ^f^	6.88 ± 0.01 ^gh^	6.40 ± 0.04 ^i^
	P1.0%		8.00 ± 0.01 ^ab^	7.42 ± 0.04 ^d^	6.95 ± 0.01 ^gh^	7.05 ± 0.04 ^fg^
	P1.5%		7.32 ± 0.00 ^de^	7.05 ± 0.06 ^fg^	7.78 ± 0.05 ^c^	6.84 ± 0.02 ^h^
Total coliforms *	Control		6.17 ± 0.24 ^abc^	5.75 ± 0.06 ^abcd^	5.91 ± 0.06 ^abc^	5.67 ± 0.01 ^abcde^
	P0.0%		6.08 ± 0.25 ^abc^	4.54 ± 0.34 ^fg^	5.78 ± 0.07 ^abcd^	5.42 ± 0.05 ^bcdef^
	P0.5%		6.76 ± 0.08 ^a^	5.60 ± 0.03 ^bcdef^	5.66 ± 0.30 ^abcde^	2.15 ± 3.04 ^g^
	P1.0%		6.43 ± 0.07 ^ab^	5.54 ± 0.04 ^bcdef^	2.30 ± 3.25 ^efg^	5.39 ± 0.18 ^bcdefg^
	P1.5%		5.80 ± 0.28 ^abcd^	5.17 ± 0.24 ^cdefg^	5.81 ± 0.06 ^abcd^	4.75 ± 0.21 ^defg^
*Escherichia coli* *	Control		4.05 ± 0.21 ^a^	2.65 ± 0.49 ^abcde^	3.29 ± 0.06 ^abcd^	1.30 ± 1.84 ^bcde^
	P0.0%		0.00 ± 0.00 ^f^	0.95 ± 1.34 ^de^	2.98 ± 0.44 ^abcd^	2.45 ± 0.21 ^cde^
	P0.5%		3.84 ± 0.34 ^abc^	3.97 ± 0.05 ^ab^	2.08 ± 0.11 ^de^	0.00 ± 0.00 ^f^
	P1.0%		3.75 ± 0.21 ^abc^	2.84 ± 0.08 ^abcde^	1.99 ± 0.30 ^de^	2.30 ± 0.00 ^de^
	P1.5%		4.01 ± 0.57 ^ab^	1.45 ± 0.21 ^e^	3.19 ± 0.11 ^abcd^	0.00 ± 0.00 ^f^
*Enterobacteriaceae* *	Control		3.38 ± 0.05 ^bcd^	2.80 ± 0.28 ^cd^	3.26 ± 0.67 ^bcd^	0.00 ± 0.00 ^e^
	P0.0%		2.30 ± 0.00 ^d^	3.32 ± 0.23 ^bcd^	2.45 ± 0.21 ^d^	0.00 ± 0.00 ^e^
	P0.5%		4.67 ± 0.29 ^a^	4.02 ± 0.29 ^ab^	0.00 ± 0.00 ^e^	0.00 ± 0.00 ^e^
	P1.0%		2.69 ± 0.13 ^cd^	4.07 ± 0.02 ^ab^	0.00 ± 0.00 ^e^	0.00 ± 0.00 ^e^
	P1.5%		3.79 ± 0.08 ^abc^	3.45 ± 0.05 ^bcd^	0.00 ± 0.00 ^e^	0.00 ± 0.00 ^e^

log CFU/g—logarithm of colony-forming units per gram (zero corresponds to counts lower than 10); Control (without formulation), P0.0% (film without propolis), P0.5% (film with 0.5% propolis), P1.0% (film with 1.0% propolis), and P1.5% (film with 1.5% propolis); *, ANOVA with a significant interaction term; different letters indicate significant mean differences (a–i).

**Table 2:** Results of statistical analysis of microbiological parameters.

Variables	Model			Interaction Term	Shapiro–Wilk Test	Levene Test
	R^2^	RSE	*p*-Value	*p*-Value	*p*-Value	*p*-Value
Total mesophiles	0.975	0.080	<0.001	<0.001	0.345	<0.001
Yeast and moulds	0.981	0.154	<0.001	<0.001	0.598	<0.001
Latic bacteria	0.996	0.041	<0.001	<0.001	0.899	<0.001
Total coliforms	0.767	0.790	0.007	<0.001	<0.001	<0.001
*E. coli*	0.938	0.379	<0.001	<0.001	0.1	<0.001
*Enterobacteriaceae*	0.987	0.203	<0.001	<0.001	<0.001	<0.001

R^2^—determination coefficient; RSE—residual standard error.

**Table 3:** Results of pH, *a*_w_, and cheese weight over time.

Measurement	Parameter	Days:	0	7	14	28
pH *	Control		5.55 ± 0.03 ^gh^	5.70 ± 0.03 ^de^	6.01 ± 0.04 ^a^	5.33 ± 0.04 ^i^
	P0.0%		5.89 ± 0.02 ^b^	5.44 ± 0.05 ^h^	5.75 ± 0.05 ^cd^	5.71 ± 0.03 ^cd^
	P0.5%		5.86 ± 0.01 ^b^	5.58 ± 0.02 ^fg^	5.81 ± 0.01 ^bc^	6.05 ± 0.05 ^a^
	P1.0%		5.74 ± 0.03 ^cd^	5.60 ± 0.02 ^efg^	5.55 ± 0.03 ^gh^	5.65 ± 0.04 ^defg^
	P1.5%		5.81 ± 0.04 ^bc^	5.66 ± 0.05 ^def^	5.44 ± 0.04 ^h^	5.75 ± 0.03 ^cd^
*a*_w_ *	Control		0.92 ± 0.008 ^a^	0.84 ± 0.004 ^bcd^	0.86 ± 0.005 ^bc^	0.66 ± 0.03 ^e^
	P0.0%		0.93 ± 0.008 ^a^	0.84 ± 0.005 ^bcd^	0.81 ± 0.02 ^d^	0.66 ± 0.007 ^e^
	P0.5%		0.90 ± 0.004 ^a^	0.83 ± 0.007 ^cd^	0.85 ± 0.002 ^bc^	0.67 ± 0.008 ^e^
	P1.0%		0.92 ± 0.004 ^a^	0.86 ± 0.003 ^b^	0.85 ± 0.006 ^bc^	0.62 ± 0.02 ^e^
	P1.5%		0.91 ± 0.004 ^a^	0.84 ± 0.007 ^bcd^	0.84 ± 0.008 ^bcd^	0.64 ± 0.001 ^e^
Cheese weight (g) *	Control		95.27 ± 0.13 ^d^	82.74 ± 0.10 ^k^	78.60 ± 0.09 ^n^	75.32 ± 0.05 ^p^
	P0.0%		104.31 ± 0.11 ^a^	93.98 ± 0.10 ^e^	88.77 ± 0.08 ^g^	84.69 ± 0.06 ^i^
	P0.5%		98.83 ± 0.15 ^b^	83.31 ± 0.12 ^j^	79.16 ± 0.09 ^m^	76.69 ± 0.07 ^o^
	P1.0%		96.27 ± 0.14 ^c^	86.77 ± 0.11 ^h^	80.97 ± 0.07 ^l^	76.88 ± 0.03 ^o^
	P1.5%		91.41 ± 0.12 ^f^	82.70 ± 0.08 ^k^	76.82 ± 0.07 ^o^	72.96 ± 0.04 ^q^

*, ANOVA with a significant interaction term; different letters indicate significant mean differences (a–q).

**Table 4:** Mean and standard deviations of HBA, HCA, FLAV, and TPCs concentrations for different HPMC/propolis formulations for T_7_ and T_28_.

Compounds	Parameter	Days:	7	28
HBA class (mg GA/L)	P1.25%		11.67 ± 1.39 ^e^	13.85 ± 1.39 ^e^
	P2.50%		16.29 ± 1.45 ^de^	16.71 ± 1.43 ^de^
	P5.00%		23.04 ± 1.65 ^c^	21.92 ± 1.58 ^cd^
	P7.50%		39.12 ± 2.03 ^b^	37.68 ± 1.94 ^b^
	P10.00%		92.18 ± 3.30 ^a^	89.33 ± 3.08 ^a^
HCA class (mg FA/L) *	P1.25%		9.97 ± 1.02 ^gh^	9.53 ± 1.01 ^h^
	P2.50%		12.00 ± 1.03 ^g^	9.98 ± 1.02 ^gh^
	P5.00%		20.40 ± 1.10 ^e^	17.40 ± 1.07 ^f^
	P7.50%		35.26 ± 1.22 ^c^	30.45 ± 1.18 ^d^
	P10.00%		80.14 ± 1.60 ^a^	70.28 ± 1.53 ^b^
FLAV class (mg Q/L) *	P1.25%		3.79 ± 0.03 ^j^	3.53 ± 0.03 ^i^
	P2.50%		4.64 ± 0.02 ^g^	4.13 ± 0.03 ^h^
	P5.00%		7.75 ± 0.02 ^e^	6.28 ± 0.02 ^f^
	P7.50%		13.34 ± 0.02 ^c^	11.94 ± 0.02 ^d^
	P10.00%		29.83 ± 0.02 ^a^	27.46 ± 0.02 ^b^
TPCs (mg TPC/L)	P1.25%		28.28 ± 4.18 ^j^	31.51 ± 4.31 ^i^
	P2.50%		34.90 ± 4.39 ^g^	34.56 ± 4.40 ^h^
	P5.00%		69.70 ± 5.54 ^e^	64.88 ± 5.40 ^f^
	P7.50%		108.62 ± 6.90 ^c^	101.55 ± 6.67 ^d^
	P10.00%		251.36 ± 12.60 ^a^	236.88 ± 11.62 ^b^

HBA—hydroxybenzoic acids; HCA—hydroxycinnamic acids; FLAV—flavonoids; TPCs—total phenolic compounds; GA—gallic acid; FA—ferulic acid; Q—quercetin; *, ANOVA with a significant interaction term; different letters indicate significant mean differences (a–j).

## Data Availability

Data available on request.

## Supplementary Materials

The following supporting information can be downloaded at: https://www.mdpi.com/article/10.3390/molecules29091941/s1, Figure S1: Linear relationship between total phenolic compounds (mg GAE/g) of formulations with different propolis concentrations; Table S1: Total phenolic compounds (mg GAE/g) of formulations with different concentrations of propolis.
