# Supplementary material for: Microbiological and Physicochemical Evaluation of Hydroxypropyl Methylcellulose (HPMC) and Propolis Film Coatings for Cheese Preservation
Source: Molecules. 2024 Apr 24;29(9):1941. doi: 10.3390/molecules29091941 (PMC11085808; doi:10.3390/molecules29091941)
Supplement: Supplementary file 1 [file molecules-29-01941-s001.zip › molecules-2938999-supplementary.pdf]

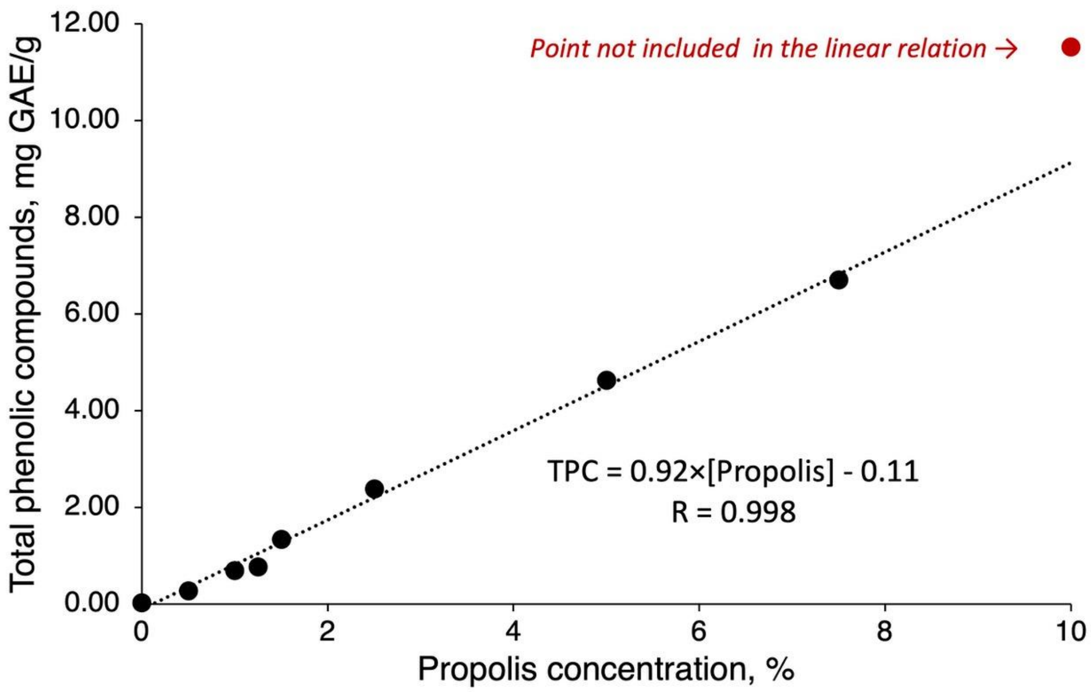

Figure S1. Linear relationship between total phenolic compounds (mg GAE/g) of formulations with different propolis concentrations.

Table S1. Total phenolic compounds (mg GAE/g) of formulations with different concentrations of propolis.

Parameter	Phenolic compounds
**P0.00%**	0.03±0.01
**P0.50%**	0.29±0.03
**P1.00%**	0.71±0.14
**P1.25%**	0.78±0.31
**P1.50%**	1.34±0.11
**P2.50%**	2.39±0.35
**P5.00%**	4.64±0.91
**P7.50%**	6.72±0.97
**P10.00%**	11.54±0.31
